# The role of psychiatrists in policy forums

**DOI:** 10.1192/j.eurpsy.2025.63

**Published:** 2025-08-26

**Authors:** M. Rojnic Kuzman

**Affiliations:** Zagreb University Hospital Centre, Zagreb, Croatia

## Abstract

**Abstract:**

On its 40th anniversary, the European Psychiatric Association (EPA) held a ceremony at the EU Parliament on December 6, 2023, and published the *Manifesto for the EU Elections* in preparation for the upcoming EU elections in June 2024.

This manifesto outlines the key goals and priorities for advancing mental health in the EU, emphasizing the vital role of psychiatrists in shaping policies and initiatives. The presentation will focus on five main topics, highlighting the involvement of psychiatrists at the European level. These include the harmonization of mental health care delivery, the improvement of working conditions and addressing the shortage of the Mental health workforce, the promotion and harmonization of ethical standards, developing new answers to an evolving World, and promotion of research and implementation of public mental health and prevention measures.

Beyond these overarching European priorities, psychiatrists are actively engaged in national policy forums, addressing a range of issues—from developing comprehensive mental health strategies to tackling specific concerns related to vulnerable populations, children, legal aspects of treatment, and more. Achieving synergy between national and international policy efforts is crucial for the successful implementation of these initiatives, which remains the core objective of this work.

**Disclosure of Interest:**

None Declared

